# Neurexin-3α IgG-mediated autoimmune encephalitis with psychiatric manifestations and choreiform movements: a case report and literature review

**DOI:** 10.3389/fimmu.2026.1944895

**Published:** 2026-10-08

**Authors:** Han Yao Shen, Xin Chen, Run Ze Yu, Wei Jie Wu, Xing Ying Dou, Xing Juan Fan

**Affiliations:** Department of Neurology, Affiliated Hospital of Nantong University, Nantong, China

**Keywords:** autoimmune encephalitis, case report, choreiform movements, neurexin-3α antibody, podocytopathy

## Abstract

**Objective:**

To summarize the clinical manifestations, ancillary examination findings, treatment course, and prognosis of a patient with neurexin-3α IgG-mediated autoimmune encephalitis presenting with prominent psychiatric disturbances and choreiform movements, and to review the literature on the clinical spectrum of this disease.

**Case summary:**

A 52-year-old woman with a history of hypertension and biopsy-proven podocytopathy with a “full-house” immunofluorescence pattern showing diffuse positivity for IgG, IgA, IgM, C3, and C1q, diagnosed in February 2026; however, a definitive diagnosis of lupus podocytopathy remained uncertain, as the patient lacked systemic lupus erythematosus manifestations. She was admitted on April 3, 2026, with a 5-day history of psychiatric disturbances and involuntary choreiform movements in all four limbs, preceded by significant psychological stress (bereavement). Neurological examination revealed slowed responses and impaired calculation. Cranial MRI showed only bilateral periventricular small ischemic foci (Fazekas grade 1) with normal SWI. Initial EEG demonstrated sharp waves and spike-and-wave complexes in the left parietal, occipital, and temporal regions. CSF analysis revealed leukocytosis (21/μl, 90% mononuclear cells), protein of 0.4 g/L, elevated IgG index of 0.76, and elevated 24-hour intrathecal IgG synthesis rate of 5.63 mg/dl. CSF mNGS detected 453 EBV sequences (RPM) with a relative abundance of 97.48%. Serum EBV VCA-IgG and EBNA-IgG were elevated with negative IgM. Autoantibody testing followed a stepwise approach: initial screening for conventional autoimmune encephalitis, paraneoplastic, and demyelinating antibodies was negative; subsequent tissue-based assay (TBA) of CSF revealed strong positive immunoreactivity, prompting expanded antibody panel testing, which ultimately detected anti-neurexin-3α antibodies in serum (titer 1:30) and CSF (titer 1:100), confirmed by both cell-based assay (CBA) and TBA. The patient’s condition progressively deteriorated, requiring endotracheal intubation on April 21 and tracheostomy on April 30. She received methylprednisolone pulse therapy at 500 mg/day for 5 days followed by a stepwise taper, intravenous immunoglobulin at 0.4 g/kg for 5 days, and five sessions of plasma exchange from April 30 to May 8 due to poor initial response. Choreiform movements gradually resolved after plasma exchange. She was weaned from mechanical ventilation on May 19 and decannulated on May 27. During the ICU course, she developed pulmonary infection with carbapenem-resistant Acinetobacter baumannii, which was controlled after antibiotic adjustment. Repeat EEG normalized on June 1. She was discharged from rehabilitation on June 9 on oral methylprednisolone 32 mg/day. On June 26, 2026, follow-up serum TBA showed weakly positive immunoreactivity, with the serum antibody titer remaining at 1:30. She subsequently received four infusions of efgartigimod at 400 mg each for consolidation immunotherapy and was transitioned to ofatumumab for long-term maintenance. At follow-up on August 6, 2026, the patient had no residual psychiatric symptoms or choreiform movements, with muscle strength recovered to 4+ in all limbs, full independence in daily activities, ambulation without assistance, and only mild gait instability.

**Conclusion:**

Neurexin-3α IgG-mediated autoimmune encephalitis is a rare but treatable condition. Choreiform movements expand its clinical spectrum. The coexistence of podocytopathy raises a hypothesis of shared antigenicity between renal and neuronal neurexin family members, though this remains purely speculative. CSF EBV reactivation may represent a bystander phenomenon or epiphenomenon of severe immune dysregulation rather than a confirmed trigger. Tissue-based assay provides crucial diagnostic direction when conventional screening is negative. Early recognition, comprehensive immunotherapy, and meticulous ICU management are important determinants of favorable outcomes. Given the inherent limitations of a single case report, these findings warrant confirmation through larger cohort studies.

## 1 Introduction

In recent years, advances in antibody detection technologies have substantially expanded the recognizable spectrum of autoimmune encephalitis. This group of disorders is mediated by autoantibodies targeting synaptic proteins or neuronal cell-surface antigens, typically presenting with acute or subacute onset of cognitive impairment, psychiatric manifestations, seizures, and altered consciousness (1). Different antibody subtypes exhibit considerable heterogeneity in their clinical presentations, tumor associations, and responses to immunotherapy (2).

Among these antibodies, anti-neurexin-3α represents a recently identified rare target. In 2016, Gresa-Arribas et al. first identified this antibody in the serum and cerebrospinal fluid of five patients with encephalitis of unknown etiology using cell-based assays, establishing its association with autoimmune encephalitis (3). Neurexin-3α is a presynaptic adhesion molecule belonging to the neurexin family, which plays a critical role in synapse development, organization, and neurotransmitter secretion (4). The proposed pathogenesis involves antibody-mediated suppression of neurexin-3α expression, leading to reduced synaptic density and disrupted balance between excitatory and inhibitory synapses in different CNS regions (3, 5). To date, 17 cases of neurexin-3α antibody-mediated encephalitis have been reported worldwide. The clinical features include a high prevalence of prodromal infections, seizures, and consciousness disturbances, with heterogeneous MRI findings and variable responses to immunotherapy (3, 6–25).

We report a case of neurexin-3α IgG-mediated encephalitis in a 52-year-old woman presenting with prominent psychiatric symptoms and choreiform movements, occurring in the context of biopsy-proven podocytopathy with a “full-house” immunofluorescence pattern (SLE uncertain). This case expands the movement disorder phenotype of neurexin-3α encephalitis and raises the hypothesis of epitope spreading or shared antigenicity between renal glomerular podocyte antigens and neuronal neurexin-3α. Cerebrospinal fluid EBV reactivation may also represent a potential trigger. The diagnostic process followed a stepwise strategy—negative conventional antibody screening prompted tissue-based assay, which provided strong positive clues that guided expanded antibody panel testing and ultimately led to the definitive diagnosis. This diagnostic approach has important clinical implications.

## 2 Case presentation

### 2.1 Clinical information

A 52-year-old woman was admitted to our hospital on April 3, 2026, with a 5-day history of psychiatric disturbances and involuntary movements.

Presenting illness: The patient developed psychiatric symptoms characterized by hyperkinesia of all limbs, emotional lability, and insomnia. She responded to questions appropriately but with slowed reactions. Her family reported that the onset was temporally related to the recent death of her mother, which had caused significant psychological distress. She had no fever, cough, sputum production, abdominal pain, diarrhea, chest tightness, dysphagia, or weight loss.

Past medical history: The patient had a history of hypertension and a diagnosis of podocytopathy confirmed by renal biopsy at our nephrology department in February 2026. Renal pathology demonstrated a “full-house” immunofluorescence pattern (positive for IgG, IgA, IgM, C3, and C1q). A definitive diagnosis of lupus podocytopathy remained uncertain, as the patient had no systemic lupus erythematosus manifestations such as arthralgia, malar rash, photosensitivity, or serositis.

Physical examination: Vital signs were stable with a temperature of 36.8 °C, pulse of 79 beats/min, respiration of 17 beats/min, and blood pressure of 120/77 mmHg. Neurological examination revealed that the patient was alert and verbally fluent but exhibited slowed responses and impaired calculation. Formal MMSE and MoCA assessments could not be performed because the patient was unable to cooperate due to psychiatric symptoms and choreiform movements. Orientation was intact. Cranial nerves were intact. Involuntary choreiform movements were observed in all four limbs, interfering with motor examination. Sensory and coordination testing could not be reliably assessed. Plantar responses were flexor bilaterally. No meningeal signs were present.

### 2.2 Complementary examinations

Routine laboratory findings: Complete blood count showed leukocytosis of 10.94×10^9^/L with 83.9% neutrophils, 13.2% lymphocytes, and 2.7% monocytes. Inflammatory markers were elevated with an hs-CRP 13.2 mg/L and ESR of 26.0 mm/h. Liver and renal function were within normal limits. Troponin, coagulation parameters, vitamin B12, plasma ammonia, urinalysis, stool examination, ASO, RF, ceruloplasmin, urinary Bence-Jones protein, parathyroid hormone, ANCA, anticardiolipin antibodies, and glycated hemoglobin were all normal.

Tumor markers (CEA panel): CA125 was elevated at 126.1 U/ml, CA724 was elevated at 31.4 U/ml, with all other markers within normal limits.

Rheumatologic markers (ENA+ANA panel): The antinuclear antibody (ANA) titer was 10.1 AU/mL (reference <32), which is considered negative; the anti-dsDNA antibody was mildly elevated at 24.6 IU/mL. Complement levels were significantly reduced, with C3 at 0.51 g/L and C4 at 0.12 g/L. The 24-hour urinary protein excretion was 4.75 g/day, reaching the nephrotic range. Urinary N-acetyl-β-D-glucosaminidase (NAG), α1-microglobulin, and β2-microglobulin were also elevated, indicating concurrent tubular interstitial involvement. All other markers within normal limits.

Thyroid function and antibodies: TSH was decreased at 0.09 mIU/L, and thyroglobulin was elevated at 83.98 mg/ml, with all other parameters within normal limits. Importantly, anti-thyroid peroxidase (TPO), anti-thyroglobulin (TG) antibodies and anti-thyrotropin receptor (TR) antibodies were both within normal limits.

Muscle enzyme panel: Creatine kinase was mildly elevated at 167 U/L, and α-hydroxybutyrate dehydrogenase was elevated at 288 U/L.which normalized upon subsequent follow-up.

Lymphocyte subsets: B lymphocytes were elevated at 29.51%, NK cells were decreased at 2.01%, total B lymphocyte count was elevated at 656 cells/μl, and NK cell count was decreased at 45 cells/μl, with all other parameters within normal limits.

Vitamin panel: Homocysteine was elevated at 10.28 μmol/L, and vitamin B6 was decreased at 1.46 ng/ml, with all other parameters within normal limits.

Immunofixation electrophoresis: Normal.

Imaging studies:

- Brain MRI: T2-FLAIR and DWI sequences showed only bilateral periventricular small ischemic foci of Fazekas grade 1, with no abnormalities on SWI and no obvious mass or signal abnormalities.
- Chest and abdominal CT (current admission): Small ground-glass opacity in the left lower lobe, heterogeneous density of the right thyroid lobe, small hepatic cyst, mild cholecystitis, and slight thickening of the left adrenal gland, with no definite space-occupying lesions.
- Renal multimodal imaging (February 2026): Bilateral renal cysts, bilateral perirenal exudation, a small amount of ascites, abdominal wall subcutaneous exudation, cholecystitis, and bilateral pleural effusion.
- Uterine ultrasound (February 2026): Uterine atrophy, uterine fibroids, normal IUD position, bilateral ovaries not well visualized, and pelvic fluid collection.
- Upper and lower extremity vascular ultrasound (February 2026): No significant abnormalities.
- Renal electron microscopy and Immunofluorescence (biopsy, February 2026): Most of the glomerular capillary basement membranes were thinner than normal, with diffuse fusion of podocyte foot processes, and a small amount of electron-dense deposits in the mesangial area. Immunofluorescence pattern, combined with light microscopy and electron microscopy, supports the diagnosis of podocytopathy (**Figure 1**).

**Figure 1:**
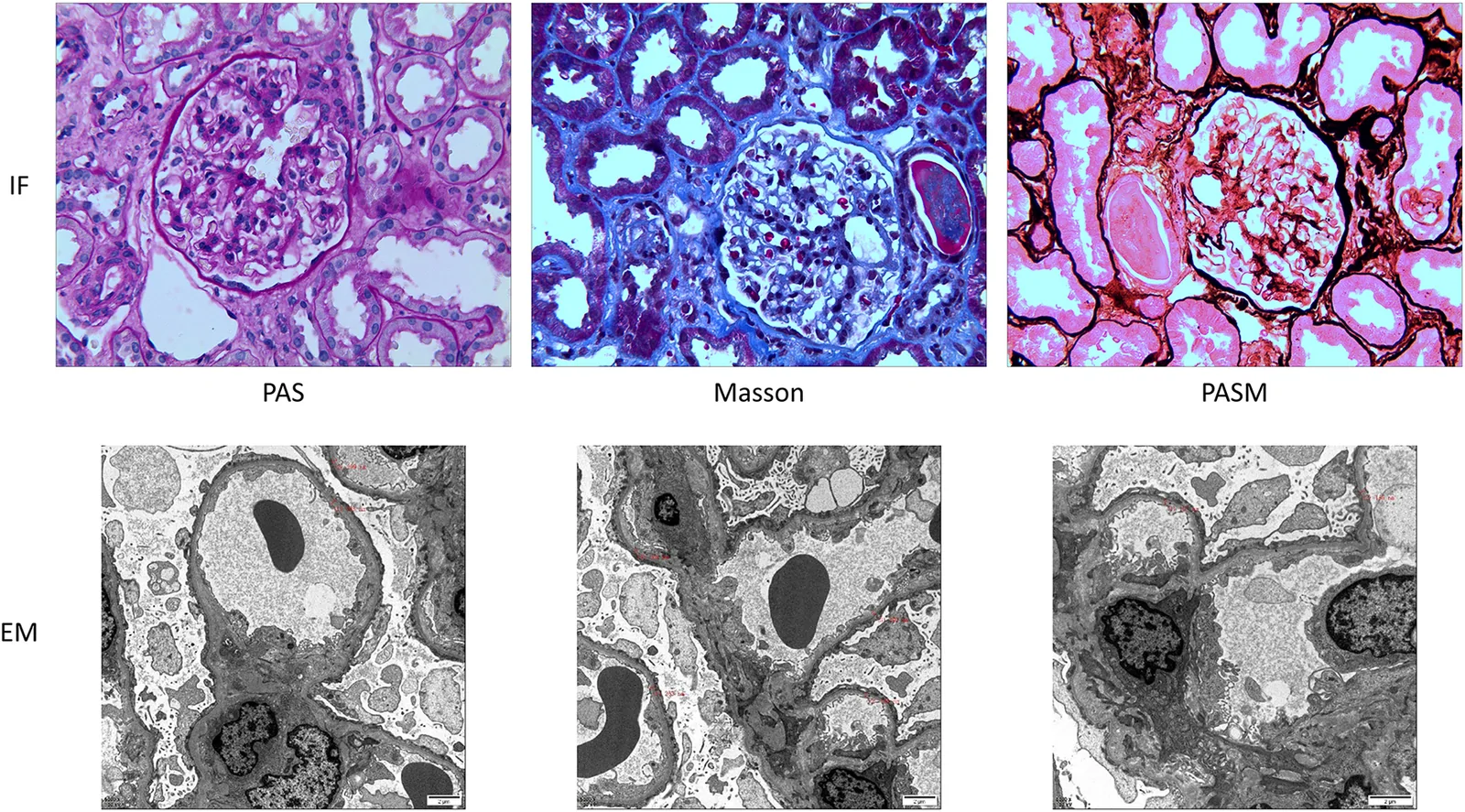
Renal pathological findings of the patient. Immunofluorescence (IF) staining demonstrated granular deposition of multiple immune complexes (including IgA, IgM, IgG, C1q, C3c, κ, and λ light chains) in the mesangial areas and capillary loops. Transmission electron microscopy (EM) revealed one glomerulus with generally patent capillary loops and focal segmental adhesions, without obvious endothelial proliferation. The glomerular basement membrane (GBM) was predominantly thin (approximately 50–60% of the loops <250 nm), with the thinnest portions measuring approximately 120–140 nm and the thickest measuring approximately 300 nm. Segmental mesangial cell proliferation and matrix expansion were present. Podocytes exhibited vacuolar degeneration, diffuse foot process effacement (>80%), and microvillous transformation, with no crescent formation observed. A few scattered electron-dense deposits were present in the mesangial areas. The renal tubules showed epithelial vacuolar degeneration, with mild focal interstitial inflammatory infiltration. Red blood cells were present within the capillary lumens. (Scale bars = 2 μm).

Electroencephalography: Initial EEG was abnormal, demonstrating sharp waves, spike-and-wave complexes, and spike-slow-wave complexes in the left parietal, occipital, and temporal regions, with generalized slowing of the background rhythm. Follow-up EEG on June 1, 2026 (after decannulation), was normal.

Lumbar puncture:

CSF was clear and colorless with normal opening pressure. CSF leukocyte count was 21/μl with 10% polymorphonuclear cells and 90% mononuclear cells. CSF chemistry: chloride 121 mmol/L, protein 0.4 g/L, and glucose 4.4 mmol/L. Simultaneous blood glucose was 7.0 mmol/L; electrolytes showed potassium 3.2 mmol/L (slightly decreased), sodium 137 mmol/L, and chloride 100 mmol/L.

CSF IgG was 4.46 mg/dl, serum IgG 960 mg/dl, serum albumin 3660 mg/dl, and CSF albumin 22.4 mg/dl. The CSF IgG index was elevated at 0.76, and the 24-hour intrathecal IgG synthesis rate was elevated at 5.63 mg/dl. CSF Gram stain, acid-fast stain, India ink stain, and routine viral antibodies were negative.

Cytokine panel (twelve cytokines): Blood cytokines showed no significant abnormalities; CSF IL-8 was markedly elevated at 140.62 pg/ml, with all other CSF cytokines within normal limits.

Metagenomic next-generation sequencing of CSF: Metagenomic next-generation sequencing of CSF revealed 453 reads per million (RPM) of Epstein-Barr virus sequences, accounting for a relative abundance of 97.48% among the detected viral reads.

Serum EBV antibodies: EBV IgG was elevated; VCA-IgG was elevated at 731.5 U/ml, EBNA-IgG was elevated at 250.6 U/ml, and EBV IgM was within normal limits.

Autoantibody testing (stepwise strategy):

Step 1 — Conventional antibody screening: Serum and CSF were screened using cell-based assays with immunofluorescence double staining for a comprehensive panel of conventional autoimmune encephalitis-associated antibodies, paraneoplastic antibodies, and inflammatory demyelinating antibodies, including: anti-NMDA receptor antibody IgG, anti-AMPA2 receptor antibody IgG, anti-LGI1 antibody IgG, anti-CASPR2 antibody IgG, anti-GlyR1 antibody IgG, anti-GABAB receptor antibody IgG, anti-GAD65 antibody IgG, anti-metabotropic glutamate receptor 5 antibody IgG, anti-AGO antibody, anti-GFAP antibody, anti-GluK2 antibody, anti-IgLON5 antibody IgG, anti-AK5 antibody, anti-mGluR1 antibody IgG, anti-KLHL11 antibody, anti-MOG antibody, anti-AQP4 antibody, anti-Hu antibody, anti-Yo antibody IgG, anti-Ri antibody IgG, anti-CV2 antibody, anti-Amphiphysin antibody, anti-Tr (DNER) antibody, anti-Recoverin antibody, and anti-SOX1 antibody. All screening results were negative.

Step 2 — Tissue-based assay: Given the negative conventional antibody results despite high clinical suspicion of autoimmune encephalitis, tissue-based assay was performed on CSF using rat brain tissue. Results demonstrated strong positive immunoreactivity with neuronal membrane antibody-positive fluorescence in the hippocampus, cerebellum, and cerebral cortex, suggesting the presence of unknown neuronal surface antibodies (**Figure 2**).

**Figure 2:**
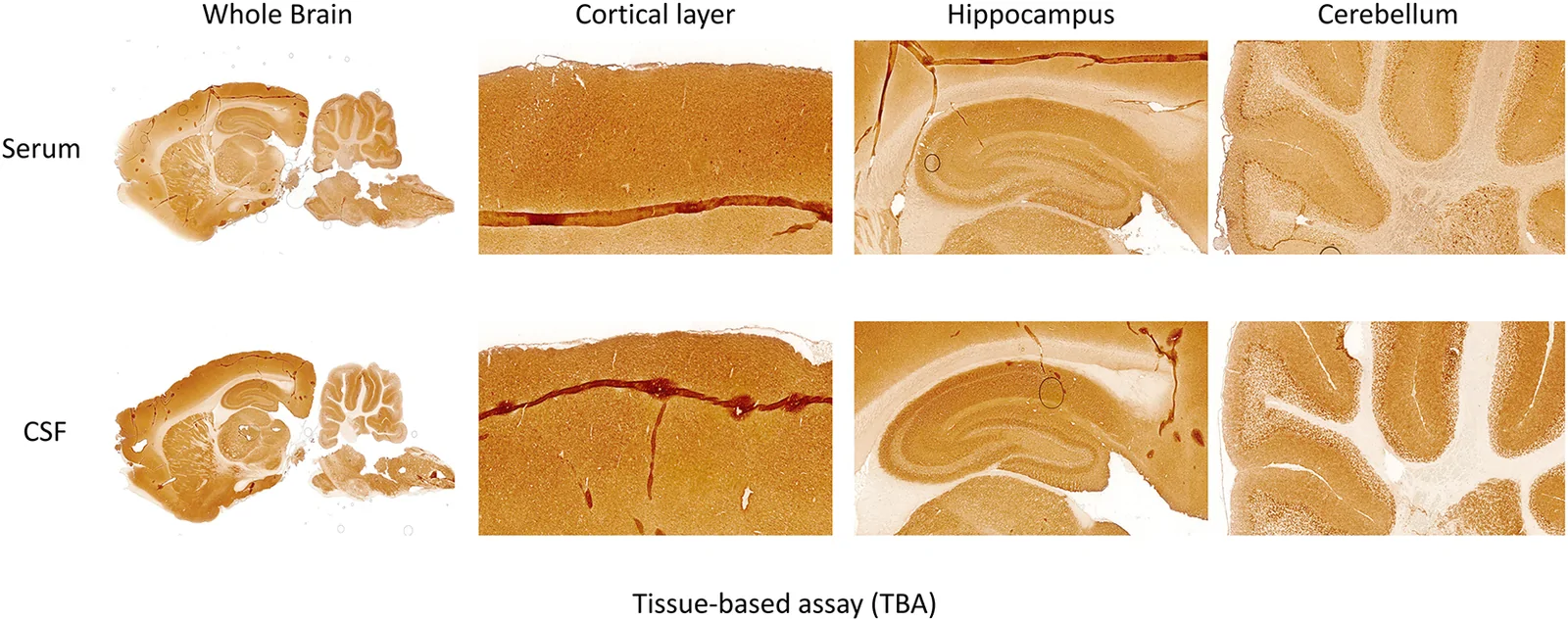
Tissue-based assay (TBA) demonstrating neurexin-3α antibody immunoreactivity in the patient’s serum and CSF. The upper panel displays staining with the patient’s serum, and the lower panel with the patient’s CSF, across whole brain, cortical layer, hippocampus, and cerebellum sections. Rat brain tissue showed prominent brown staining with a characteristic neuropil-like pattern. Positive immunoreactivity was predominantly localized to the molecular and granular layers of the cerebellum, the pyramidal layer and radiatum of the hippocampus, and along the pial surface/superficial cortex. This staining pattern is suggestive of antibodies targeting neuronal cell-surface antigens.

Step 3 — Expanded antibody panel testing: Based on the strong positive tissue-based assay findings, the autoantibody panel was further expanded, ultimately detecting positive anti-neurexin-3α antibodies in both serum (titer 1:30) and CSF (titer 1:100), confirmed by both cell-based assay and tissue-based assay (**Figure 3**). All other tested antibodies were negative.

**Figure 3:**
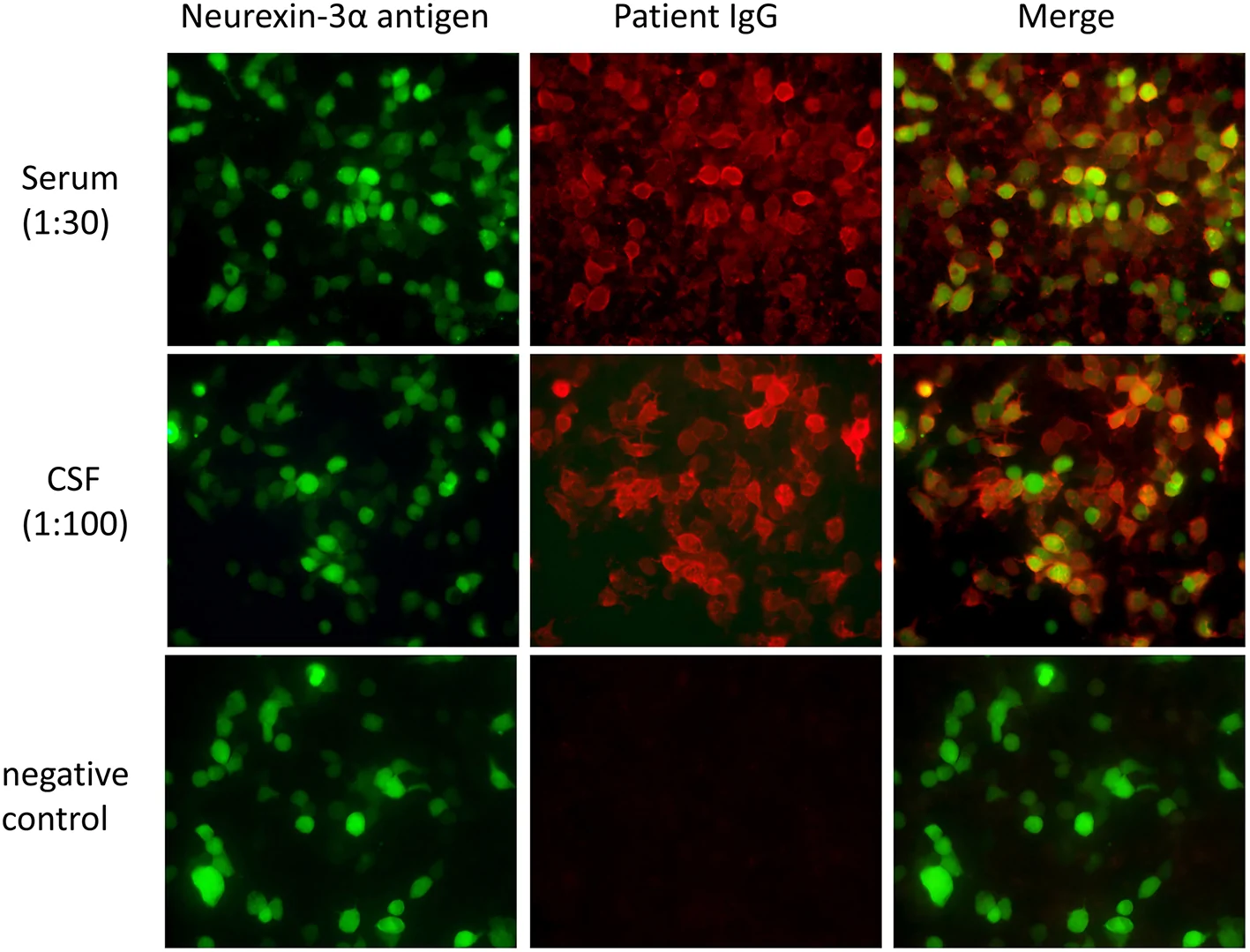
Cell-based assay (CBA) confirming neurexin-3α antibodies in the patient’s serum and CSF. HEK293T cells expressing neurexin-3α antigen were incubated with patient samples. The left column shows the neurexin-3α antigen (green fluorescence), the middle column shows patient IgG binding (red fluorescence), and the right column displays merged images. Co-localization of patient IgG and the neurexin-3α antigen appears as yellow/orange. Both the serum (1:30 dilution) and CSF (1:100 dilution) samples demonstrated positive immunoreactivity. The negative control showed only green antigen signals without red patient IgG binding. However, due to the lack of paired titration to calculate the specific antibody index, definitive intrathecal synthesis could not be formally established in this case.

To ensure full methodological transparency, the detailed procedures and interpretation criteria for the antibody assays are described as follows. The testing was performed at KingMed Diagnostics (Nanjing, China). For cell-based assays (CBA), HEK293T cells were transfected with the target antigen and green fluorescent protein (GFP) as an internal control. Antibody positivity was defined as clear red fluorescence localized to the cell membrane of successfully transfected cells under the red channel, confirmed by co-localization with the green signal in the merged channel. Both standard negative controls and GABABR/LGI1 positive controls were included in each experiment. For the tissue-based assay (TBA), rat brain sections were used as the substrate, and staining was visualized using DAB chromogen. Serum samples were tested at a dilution of 1:200, while CSF samples were tested at 1:1. Positive TBA results were defined as distinct brown staining in the neuropil of the hippocampus, cerebral cortex, and cerebellum (involving the molecular layer, granular layer, and Purkinje cells) that was significantly stronger than the negative controls. The characteristic neuropil-like pattern observed in our patient supports the presence of antibodies against neuronal cell-surface antigens.

### 2.3 Treatment and follow-up

Initial treatment: On April 9, 2026, prior to the availability of metagenomic next-generation sequencing results, the patient was started on oral methylprednisolone, haloperidol for psychiatric symptom control, sodium valproate and levetiracetam for seizure prophylaxis, and intravenous ganciclovir. The rationale for ganciclovir was based on the initial CSF findings of a mild leukocytic pleocytosis (21 cells/μl) with mononuclear predominance and normal glucose and chloride levels. This pattern raised the suspicion of a potentially treatable viral encephalitis, such as a herpesvirus infection, especially given the patient’s underlying immune dysregulation. Therefore, ganciclovir was empirically administered as a pre-emptive anti-viral measure while the differential diagnosis between infectious and autoimmune encephalitis was being investigated. Its specific therapeutic contribution to the patient’s ultimate clinical recovery remains unclear, given that the definitive diagnosis was ultimately anti-neurexin-3α autoimmune encephalitis treated with immunomodulatory therapies. Diazepam, midazolam, and dexmedetomidine were used intermittently for sedation.

After confirmation of neurexin-3α antibody positivity on April 15, she received intravenous methylprednisolone pulse therapy at 500 mg/day for 5 days, followed by stepwise tapering: 250 mg/day for 3 days, 120 mg/day for 5 days, 60 mg/day for 7 days, then transitioned to oral prednisone 56 mg/day with weekly reduction of 4 mg. Intravenous immunoglobulin was administered at 0.4 g/kg/day for 5 consecutive days (April 16–20).

Phase 1: Respiratory support and endotracheal intubation (April 21–29, 2026)

On April 21, 2026, the patient developed respiratory distress, cyanosis, oxygen desaturation, and blood pressure instability. After discussion with the family, endotracheal intubation was performed and mechanical ventilation was initiated in IMV mode: FiO_2_ 45%, VT 420 ml, respiratory rate 14 breaths/min, PSV 8 cmH_2_O, PEEP 5 cmH_2_O. Deep sedation and analgesia (midazolam, remifentanil) were administered to control agitation and reduce oxygen consumption, and norepinephrine was given for blood pressure support.

From April 22 to 29, the patient remained sedated with intermittent agitation and fluctuating GCS scores. She developed recurrent fever (peak 38.3 °C) with elevated infection markers. On April 27, due to poor spontaneous breathing and airway protection, weaning and extubation were deemed difficult, and tracheostomy was planned. On April 28, the necessity and risks of tracheostomy were communicated with the family, and plasma exchange was simultaneously prepared.

Phase 2: Tracheostomy, plasma exchange, and anti-infection therapy (April 30 – May 17, 2026)

On April 30, 2026, ultrasound-guided percutaneous tracheostomy was performed at the 2nd–3rd tracheal cartilage ring space. The procedure was uneventful without significant bleeding. The tracheostomy tube was properly secured and connected to mechanical ventilation. The corticosteroid dose was reduced to 60 mg/day, and the first session of plasma exchange was performed on the same day.

On May 1, the patient continued to have fever (peak 38.8 °C). Sputum culture returned positive for carbapenem-resistant *Acinetobacter baumannii*. On May 2, the antibiotic regimen was adjusted to cefoperazone-sulbactam 2g Q8h (8-16-24) plus tigecycline 100mg Q12h.

Plasma exchange was performed on April 30, May 2, May 4, May 6, and May 8, for a total of 5 sessions.

From May 9 to 17, the patient gradually developed scattered ecchymoses, a rash extending from the posterior neck to the buttocks, and bilateral oral herpes, suggestive of infection-related rash and possible viral reactivation. On May 14, redness was noted at the left internal jugular central venous catheter insertion site, prompting enhanced local wound care and vigilance for catheter-related bloodstream infection. With continued anti-infective and immunomodulatory therapy, the patient’s fever resolved by May 17, agitation significantly improved, and infection markers trended downward.

Phase 3: Weaning from ventilation and airway closure (May 18–27, 2026)

On May 18, the patient’s consciousness improved, spontaneous breathing recovered well, and inflammatory markers decreased significantly. Weaning assessment was initiated with gradual reduction of ventilatory support.

From May 19 to 20, the patient was successfully weaned from mechanical ventilation and transitioned to oxygen delivery via tracheostomy tube at 3 L/min, with stable breathing and vital signs. GCS improved to 11, with clear consciousness and appropriate verbal responses.

On May 25, after active airway management including humidification and chest physiotherapy, respiratory secretions decreased and spontaneous breathing was robust. To assess the patient’s ability to breathe via the upper airway, the tracheostomy tube was changed to a metal tube with occlusion. The patient tolerated the occlusion well with effective spontaneous coughing.

On May 27, after successful tube occlusion for over 48 hours without respiratory distress and with stable oxygen saturation, the tracheostomy tube was successfully removed, and the wound was covered with sterile dressing after local disinfection. The patient maintained stable breathing without local bleeding or respiratory distress. Clinical notes on May 29 documented the tracheostomy site covered with sterile dressing without bleeding or exudate; on May 30, the wound was healing well without redness, swelling, or exudate.

Phase 4: Rehabilitation and subsequent immunotherapy (June 4 – July 6, 2026)

On June 1, 2026, follow-up EEG was normal.

On June 4, the patient was transferred from the intensive care unit to the rehabilitation medicine department for comprehensive rehabilitation therapy, including limb function training, electrical stimulation physiotherapy, and respiratory training, due to residual limb weakness and motor impairment.

On June 9, the patient progressed well in rehabilitation, with clear consciousness, stable mental status, muscle strength recovered to grade 4+ in all limbs, sitting and standing balance reaching grade 3, and bilateral pathological reflexes negative. She was discharged from the rehabilitation department on oral methylprednisolone 32 mg/day with continued weekly tapering of 4 mg and was advised to follow up at the neurology and endocrinology departments. The discharge summary documented the tracheostomy site healing well without bleeding, exudate, or redness.

On June 26, 2026, the patient returned for follow-up serum TBA and antibody titer testing. TBA showed weakly positive immunoreactivity, and the serum anti-neurexin-3α antibody titer remained at 1:30, unchanged from baseline, although TBA fluorescence intensity had markedly decreased compared with the strong positive result at initial diagnosis.

On June 29 and August 6, the patient received the first two intravenous infusions of efgartigimod at 400 mg each for consolidation immunotherapy. Due to the favorable clinical response and excellent tolerability, she received two additional infusions of efgartigimod at 400 mg each in the subsequent weeks, totaling four doses. Both the transient FcRn antagonism and the initial plasma exchange served as bridging strategies during the acute phase, which was characterized by severe infection risk. Once her overall condition stabilized and the risk of severe infection markedly decreased, she was transitioned to regular subcutaneous injections of ofatumumab, an anti-CD20 monoclonal antibody, for long-term maintenance immunotherapy. She is currently on a stable regimen of ofatumumab without any reported adverse events, and her neurological status remains stable with no recurrence.

Follow-up (August 6, 2026): At the 3-month follow-up, the patient had no residual psychiatric symptoms or choreiform movements. Muscle strength recovered to 4+ in all limbs, bilateral pathological reflexes were negative, and she was fully independent in daily activities, could walk without assistance, and exhibited only mild gait instability.

Clinical Timeline:

The patient’s clinical course is summarized chronologically as follows:

February 2026: Biopsy-proven podocytopathy was diagnosed.

Late March 2026: Psychological stress (bereavement) occurred, followed by the gradual onset of psychiatric symptoms.

April 3, 2026: The patient was admitted to our hospital with prominent psychiatric disturbances and choreiform movements.

April 9, 2026: Initial empiric treatment was initiated with oral methylprednisolone, antiepileptic drugs, and intravenous ganciclovir.

April 15, 2026: Anti-neurexin-3α antibodies were confirmed (serum 1:30, CSF 1:100). High-dose methylprednisolone pulse therapy was started.

April 16-20, 2026: Intravenous immunoglobulin was administered.

April 21, 2026: The patient developed respiratory failure and required endotracheal intubation and ICU admission.

April 30, 2026: Tracheostomy was performed.

April 30 – May 8, 2026: Five sessions of plasma exchange were completed.

May 1–17, 2026: Carbapenem-resistant Acinetobacter baumannii pneumonia was treated and controlled.

May 27, 2026: The patient was successfully decannulated.

June 1, 2026: Follow-up EEG normalized.

June 9, 2026: The patient was transferred to the rehabilitation department and subsequently discharged.

June 26, 2026: Serum TBA became weakly positive, with the serum antibody titer remaining at 1:30.

June 29 – August 6, 2026: Received four infusions of efgartigimod as bridging therapy and transitioned to ofatumumab for long-term maintenance.

August 6, 2026: Clinical recovery was achieved, with muscle strength of 4+ and no residual psychiatric or choreiform symptoms.

## 3 Discussion

Neurexin-3α is a presynaptic cell-adhesion molecule belonging to the neurexin family, which plays an essential role in synapse development, organization, and neurotransmitter secretion (4). The pathogenesis of neurexin-3α antibody-associated encephalitis is thought to involve antibody-mediated suppression of neurexin-3α expression, leading to reduced synaptic density and disrupted balance between excitatory and inhibitory synapses in different CNS regions (3, 5). This mechanism may account for the wide spectrum of clinical manifestations, including seizures, cognitive decline, psychiatric symptoms, and movement disorders.

Including our case, a total of 18 patients with neurexin-3α antibody-mediated encephalitis have been reported to date (**Table 1**). Among these, 9 were female and 9 were male, with ages ranging from 18 to 74 years (median: 44 years). Antecedent infection-like prodromal symptoms were present in 13 of 18 patients (72.2%), most commonly fever, headache, and gastrointestinal symptoms. Seizures and decreased consciousness were the most frequent manifestations, occurring in 16 of 18 patients (88.9%). Psychiatric symptoms, including confusion, agitation, and hallucinations, were reported in 13 of 18 patients (72.2%). Movement disorders were described in only a minority of cases, and choreiform movements, as seen in our patient, have been rarely highlighted—only mild orofacial dyskinesia was mentioned in case 3 and case 4 of the original series by Gresa-Arribas et al. (3). Our case thus adds a distinctive feature to the movement disorder phenotype of this disease.

**Table 1:** Main clinical data of reported patients with neurexin-3a antibody-mediated encephalitis (n = 16).

Patient	Sex	Age (years)	Prodromal symptoms	Main clinical manifestations	MRI	CSF findings	Treatment	Prognosis	Other characteristics
1 (3)	Female	23	Headache	Confusion, drowsiness, memory impairment, and suspected central hypoventilation	Normal	First: normal Second: white blood cell count 20	Corticosteroids	Followed up after 36 months: partial recovery of symptoms, residual memory deficits, anxiety, nocturnal bilevel positive airway pressure ventilation	Complicated with leukopenia, transient thrombocytopenia
2 (3)	Male	23	Headache, fever	Blurred consciousness, extreme agitation, generalized spasms, myoclonus, coma	Normal	White blood cell count: 31	Corticosteroids	No effect of treatment; died on day 17	Accompanied by thrombocytopenia, leukopenia, antinuclear antibodies of 1:60; autopsy suggested cerebral edema and brain herniation
3 (3)	Female	50	Headache, fever, vomiting, diarrhea	Abnormal behavior, seizures, decreased level of consciousness, mild orofacial dyskinesia, central hypopnea	Abnormal signal of FLAIR/T2 and DWI in the medial temporal lobe	Increased IgG index	corticosteroids, cyclophosphamide	Followed up after 4 months: partial recovery of symptoms, residual cognitive impairment, and refractory epilepsy	History of systemic lupus erythematosus and Raynaud’s disease
4 (3)	Female	44	Headache, fever	Confusion, agitation, seizures, incoherent speech, mild orofacial dyskinesia	Normal	10 white blood cells	Corticosteroids, immunoglobulin	Died after the 67th day of septicemia	Antinuclear antibody of 1:1 280; autopsy showed mild subarachnoid hemorrhage, and lung showed cytomegalovirus infection
5 (3)	Female	44	Nausea, diarrhea	Confusion, memory impairment, seizures	Normal	24 white blood cells	Corticosteroids	Followed up after 2 months: significant recovery of symptoms, residual mild cognitive impairment (still improving)	With chronic arthralgia, positive thyroglobulin antibodies
6 (8)	Male	57	Fever, fatigue, dizziness, muscle pain	Apathy, drowsiness, interrupted sleep- wake cycle, confusion and behavioral changes	Slightly high signal in the caudate nucleus- inner capsule gap	159 cell counts (91% monocytes)	Corticosteroids	Complete relief of symptoms after 15 days	Diagnosis of Plasmodium falciparum infection prior to onset of disease
7 (9)	Male	58	None	Seizures, memory loss, depressed mood, psychomotor retardation	Abnormal signal in the left temporal lobe	Elevated levels of phosphorylated tau protein (70 pg/mL)	Corticosteroids	No apparent change	Positive Yo and SOX1 antibodies; 18F-FDG-PET/CT showed marked hypometabolism in the bilateral prefrontal and parietal lobes
8 (17)	Female	59	Non-specific prodromal symptoms, not detailed	Blurred consciousness, decreased concentration, disorientation, seizures	Increased volume of the left hippocampus, T2 high signal	Mild cellular proliferation	Corticosteroids, plasma exchange, rituximab	Complete remission on day 29	History of rheumatoid arthritis, Hashimoto’s thyroiditis
9 (18)	Male	56	Fever, cough	Memory loss, seizures, myoclonic seizures, involuntary movements, dystonia, autonomic abnormalities	FLAIR high signal in the bilateral temporal lobe, hippocampus, and insula	Normal at 2 times	Corticosteroids, immunoglobulin	Died 10 days later	NA
10 (19)	Female	21	NA	Progressive motor retardation, apathy and hypotonia, unsteady walking, trembling of limbs	Slight dilatation of the ventricular system, atrophic changes in the brain with widening of the sulcal pools	Leukocytes 14×106/L, protein level 498.50 mg/L, glucose 4.56 mmol/L	Corticosteroids, immunoglobulins, azathioprine	Symptoms improved after 16 days, relapsed on self- discontinuation, improved on retreatment	Psychological stimuli prior to onset of illness
11 (20)	Female	42	Headache and fever after yellow fever vaccination	Seizures, psychotic symptoms, disorders of consciousness, hallucinations, delusions of victimization, and agitation	NA	Leukocytes 15/mm3, Normal protein	Corticosteroids, immunoglobulins, azathioprine	Walking with unilateral support after three months, occasional mild paranoia of victimization, otherwise functioning normally	Positive antibodies to ANA, ds- DNA, and SSA; history of arthritis, confirmed diagnosis of SLE
12 (21)	Female	74	NA	Bilateral lower extremity weakness, cognitive impairment	Hypometabolism in the prefrontal and parietal lobes, hypermetabolism in the medial temporal lobe	NA	Plasma exchange, immunoglobulins, corticosteroids	Immunotherapy was ineffective and eventually underwent stereotactic needle brain biopsy of a right frontal lobe lesion	Pathology of the posterior frontal lobe showing diffuse large B-cell lymphoma
13 (22)	Male	54	NA	Recurrent generalized tonic–clonic seizures, impaired consciousness, psychotic symptoms (such as hallucinations), cognitive dysfunction, and a significant decrease in sleep time	Cerebral edema and meningitis, with major consideration of contrast- induced encephalopathy	Increased white blood cells and protein	Corticosteroids, MMF	Recovered	Postcarotid stenting
14 (23)	Male	18	Headache, fever	Delirium of consciousness, new-onset refractory status epilepticus (NORSE)	Normal	Increased cells, lymphocyte predominance, elevated proteins	Plasma exchange, immunoglobulins, corticosteroids	Died 19 days after admission	Lymphopenia, mild thrombocytopenia, and elevated transaminases
15 (6)	Male	43	Sore throat, runny nose, and cough	Loss of consciousness, seizures	Lesion in the frontal lobe	Leukocytes 15/mm3, lymphocytes 85%	Corticosteroids	Recovered	Antinuclear antibodies of titer 1:3200 (titer 1:3200 nuclear granular, titer 1:320 cytoplasmic granular), positive SSA/Ro52, and Sm antibodies
16 (25)	Male	55	Fever, vomiting, dizziness, headache	Visual obscuration, cephalalgia with associated emetic episodes, concurrent neuropsychiatric manifestations	Few punctate abnormal signals adjacent to the lateral ventricles, with no enhancement	30 white blood cells	Corticosteroids, immunoglobulin	Complete remission occurred on the 40th day	Plasma levels of ANA, anti-dsDNA, rheumatoid factor were increased; Plasma ANCA, anti-SS-A/RO60KD, anti-SS-A/RO52, anti-SnRNP, and lupus anticoagulant were positive; SLE diagnosed
17 (24)	Male	26	Headache and 1-week history of fever	Status epilepticus, mental and behavioral disturbances, cognitive impairment	Hyperintense signals in the bilateral hippocampus and leptomeningeal enhancement in the left cerebral hemisphere	Leukocyte count 39 cells/μL (91% polymorphonuclear), mildly elevated protein (0.64 g/L), NGS negative	Corticosteroids, IVIG, rituximab (maintenance)	Neurologically stable at 3- and 5-month follow-up, returned to work	Serum neurexin-3α titer 1:10 (CBA), serum MOG titer 1:32, confirmed by TBA
18 This case	Female	52	Psychological stress (bereavement)	Psychiatric symptoms, prominent choreiform movements, respiratory failure requiring intubation/tracheostomy	Bilateral periventricular small ischemic foci (Fazekas grade 1)	Leukocytes 21/μl, 90% mononuclear cells, elevated IgG index (0.76), 24h IgG synthesis rate 5.63 mg/dl, EBV 453 RPM (97.48% abundance)	Corticosteroids, IVIG, plasma exchange (5 sessions), efgartigimod (4 infusions), ofatumumab (maintenance)	Significant recovery, resolution of psychiatric and movement symptoms, muscle strength 4+	Biopsy-proven podocytopathy with full-house IF pattern, anti-dsDNA positive, C3/C4 low, ANA negative; all thyroid antibodies negative

Cranial MRI findings in neurexin-3α encephalitis are heterogeneous: among the 18 reported patients, 4 had normal findings, 12 showed abnormal signals in various brain regions (temporal lobe, hippocampus, caudate-capsule-lenticular regions, or frontal lobe), and 2 lacked detailed imaging data. Our patient’s MRI showed only Fazekas grade 1 periventricular microangiopathic changes with no specific encephalitis-related abnormalities, reinforcing that normal or non-specific MRI does not exclude neurexin-3α encephalitis.

CSF abnormalities were present in all 18 patients. Our patient had mild lymphocytic pleocytosis of 21/μl, 90% mononuclear cells and mildly elevated protein at 0.4 g/L. Both the CSF IgG index of 0.76 and 24-hour intrathecal IgG synthesis rate at 5.63 mg/dl were elevated, indicating active intrathecal immunoglobulin synthesis, consistent with previous reports.

The unique aspects and novel findings of our case include the following:

First, prominent choreiform movements. Our patient presented with prominent choreiform movements of all four limbs, while only mild orofacial dyskinesia was described in previous cases. This significantly expands the movement disorder spectrum of neurexin-3α encephalitis and suggests that choreiform movements should be recognized as a possible presenting feature.

Second, the stepwise diagnostic strategy for autoantibody detection. The diagnostic process in this case demonstrates a clear stepwise approach: when conventional cell-based assay screening for common autoimmune encephalitis, paraneoplastic, and demyelinating antibodies was negative, tissue-based assay provided strong positive clues that guided expanded antibody panel testing and ultimately led to the identification of the rare neurexin-3α antibody. This diagnostic pathway highlights the critical directional value of tissue-based assay in patients with high clinical suspicion of autoimmune encephalitis but negative conventional antibody results, effectively preventing missed diagnosis of rare antibody-mediated encephalitis.

Third, CSF EBV reactivation as a potential trigger. Cerebrospinal fluid metagenomic next-generation sequencing (mNGS) detected a high relative abundance of EBV sequences (453 RPM; 97.48% abundance). Serum serology showed markedly elevated VCA-IgG and EBNA-IgG with negative IgM, which is consistent with EBV reactivation rather than primary infection.

We must emphasize, however, that the RPM value obtained from mNGS is a semi-quantitative measure of relative abundance and does not constitute quantitative viral load data. Since quantitative real-time PCR was not performed in our laboratory, the absolute EBV copy number remains unknown. Given this limitation, the high relative abundance of EBV sequences may reflect a bystander phenomenon or a nonspecific consequence of severe systemic inflammation and immune dysregulation, rather than providing definitive evidence of a causal role. Therefore, the relationship between EBV reactivation and the onset of neurexin-3α encephalitis in our patient remains entirely speculative and warrants further mechanistic investigation.

EBV has been proposed as a possible trigger for autoimmune encephalitis through molecular mimicry or bystander immune activation (10, 26). Post-infectious autoimmune encephalitis with anti-NMDAR antibodies has been reported during recovery from herpes simplex virus encephalitis. Therefore, the causal relationship between EBV reactivation and the onset of neurexin-3α encephalitis in our patient remains entirely speculative. We reframing it as a possible trigger or an epiphenomenon that warrants further mechanistic investigation, and clearly acknowledging the lack of PCR data as a limitation of this case.

Fourth, markedly elevated CSF IL-8. Cerebrospinal fluid IL-8 reached 140.62 pg/ml, while blood cytokines showed no abnormalities. IL-8 is an important neutrophil chemokine that is elevated in various CNS inflammatory conditions; its significance in neurexin-3α encephalitis remains to be elucidated.

Fifth, coexistence with podocytopathy—the epitope spreading hypothesis. Our patient had biopsy-proven podocytopathy with a “full-house” immunofluorescence pattern showing diffuse deposition of IgG, IgA, IgM, C3, and C1q, elevated serum anti-ds-DNA antibody (24.6 IU/ml), yet no systemic lupus erythematosus manifestations such as arthralgia, malar rash, or serositis, making the diagnosis of SLE uncertain. Of interest, neurexin-1 (NRXN1), a closely related family member of neurexin-3α, has been shown to be expressed in renal glomerular podocytes and plays a role in podocyte architecture (27). A recent genome-wide association study identified NRXN3 as a causal gene associated with diabetic kidney disease, linking this neurexin family member to kidney pathology through immune cell infiltration pathways (28). Based on these observations, particularly the coexistence of podocytopathy and anti-neurexin-3α antibodies in the same patient—we propose a hypothesis of epitope spreading or shared antigenicity between renal podocyte antigens and neuronal neurexin-3α. Importantly, this hypothesis does not depend on a definitive diagnosis of SLE, but rather on the potential immunological cross-reactivity between glomerular podocyte antigens and neuronal neurexin family members.

However, we must emphasize that this is a purely speculative hypothesis, not a demonstrated mechanism. To our knowledge, while cases of neurexin-3α encephalitis coexisting with SLE have been reported, no previous case of neurexin-3α encephalitis arising in a patient with underlying podocytopathy has been specifically reported, and this observation warrants further investigation. Raising a hypothesis of shared antigenicity or epitope spreading between renal podocyte and neuronal neurexin antigens that warrants future investigation through targeted immunohistochemical studies and cross-reactivity assays. This association should not be interpreted as implying a causal link.

Sixth, complex ICU course and complication management. Our patient’s clinical course was particularly challenging—despite initial high-dose corticosteroids and intravenous immunoglobulin, she continued to deteriorate, requiring ICU admission for mechanical ventilation and vasopressor support. Although her choreiform movements gradually resolved after five sessions of plasma exchange, it is important to emphasize that this clinical improvement occurred concurrently with high-dose corticosteroids, IVIG, subsequent novel immunotherapies (efgartigimod and ofatumumab), and prolonged multidisciplinary rehabilitation. Therefore, the observed improvement should not be attributed solely to plasma exchange, but rather to the synergistic effects of this comprehensive multi-modal treatment strategy. During ICU support, the patient developed pulmonary infection with carbapenem-resistant Acinetobacter baumannii and catheter-related infection risk, which were controlled after antibiotic adjustment. Additionally, the patient developed generalized rash and oral herpes, suggestive of infection-related rash and possible viral reactivation. This course highlights that prevention and management of opportunistic infections in severely immunosuppressed autoimmune encephalitis patients is a critical determinant of prognosis, requiring close multidisciplinary collaboration. The EEG normalized on June 1 following decannulation, suggesting significant recovery of brain function with control of neuroinflammation and withdrawal of respiratory support.

Seventh, implications of antibody dynamics monitoring. On June 26, 2026, approximately 7 weeks after plasma exchange completion and 17 days after discharge, serum anti-neurexin-3α antibody testing showed weakly positive TBA immunoreactivity, though the antibody titer remained at 1:30, unchanged from baseline. Despite the lack of a significant decline in antibody titer, the patient’s clinical symptoms—psychiatric disturbances, choreiform movements, and consciousness impairment—had markedly improved, with muscle strength recovered to 4+ and full independence in daily activities. This dissociation between antibody titer and clinical status suggests several possibilities. First, the decrease in TBA fluorescence intensity from strongly positive to weakly positive may reflect changes in antibody properties (such as affinity or epitope specificity) or target antigen accessibility, rather than simply a reduction in antibody quantity. Second, clinical improvement likely involves not only clearance of circulating antibodies but also modulation of the CNS immune microenvironment, restoration of synaptic plasticity, recovery of blood-brain barrier integrity, and reorganization of neural functional networks (1, 3, 29, 30). Third, the patient received efgartigimod infusions on June 29 and July 6, after which muscle strength further improved from grade 4 to 4+, suggesting that FcRn antagonism may contribute to sustained benefit through continued reduction of pathogenic IgG or immunomodulatory effects; however, the exact impact on antibody titers requires longer follow-up and additional treatment courses to assess. This observation underscores that, when evaluating immunotherapy responses, clinical improvement, TBA fluorescence pattern changes, and antibody titers should be considered together rather than relying solely on titer values.

Eighth, stepwise immunotherapy and long-term maintenance. The treatment paradigm in our case involved a carefully designed stepwise approach to balance efficacy with infection risk. During the critical phase with concurrent CRAB pneumonia and herpes reactivation, we utilized plasma exchange and four infusions of efgartigimod, a selective FcRn antagonist. Efgartigimod provided a rapid, targeted reduction of pathogenic IgG without causing broad immunosuppression (31). As the patient’s infection risk subsided, we escalated to ofatumumab, an anti-CD20 monoclonal antibody that depletes B cells, to provide durable immunomodulation. This sequential therapy demonstrates that the initial lack of traditional immunosuppressant escalation was not due to treatment refractoriness, but a deliberate clinical decision to prioritize infection control before introducing classical second-line agents. This case suggests that a “bridge-and-maintain” strategy using FcRn antagonists followed by anti-CD20 therapies may be a viable and safe alternative for managing severe autoimmune encephalitis with high infectious complications.

Regarding differential diagnosis, several conditions warrant consideration in this case: (1) Lupus encephalopathy—although the patient had underlying lupus podocytopathy, the diagnosis of lupus encephalopathy requires exclusion of other etiologies (32); the presence of definite neurexin-3α antibodies supports the diagnosis of autoimmune encephalitis rather than isolated lupus encephalopathy. (2) Hashimoto’s encephalopathy—despite thyroid function abnormalities (decreased TSH, elevated thyroglobulin), other thyroid antibodies were not significantly elevated, and the presence of neuronal surface antibodies favors autoimmune encephalitis (33). (3) EBV encephalitis—CSF EBV-DNA positivity can be seen in various conditions (34); our patient had negative EBV-CA-IgM and no other pathogens detected by NGS, suggesting EBV reactivation as an immune trigger rather than active EBV encephalitis.

Interpreting the non-specific laboratory abnormalities in this case, our assessment is as follows: Regarding the thyroid dysfunction, although TSH was markedly decreased and thyroglobulin elevated, anti-TPO, anti-TG and anti-TR antibodies were both negative, which argues against the diagnosis of Hashimoto’s encephalopathy. The thyroid abnormalities were more likely attributable to non-thyroidal illness syndrome or medication effects during the acute critical illness. The transiently elevated creatine kinase was likely a consequence of the increased muscular workload from the persistent, severe choreiform movements; this notion is supported by its normalization on subsequent testing, without evidence of an underlying primary myopathy. With respect to the elevated CA125 and CA724 levels, the patient had documented bilateral pleural effusions, ascites, and pelvic fluid collection, which are well-known benign causes of elevated tumor markers. A thorough paraneoplastic workup, including whole-body CT and multi-regional ultrasound, revealed no definite malignant lesions, further supporting a benign attribution. We acknowledge that follow-up testing for these tumor markers was not performed, which is a minor limitation of this case.

Regarding the differential diagnosis of the prominent choreiform movements observed in our patient, we have systematically considered several additional categories of adult-onset chorea. Autoimmune and autoimmune-associated causes were at the forefront, including Sydenham chorea, systemic lupus erythematosus (SLE)-related chorea, and antiphospholipid antibody syndrome, which are well-recognized autoimmune causes of chorea. Other autoimmune encephalitides, such as anti-NMDAR and anti-LGI1 encephalitis, were also considered as potential mimics. Given the CSF findings, viral encephalitis was included in the differential. Metabolic and endocrine causes, including nonketotic hyperglycemia, hyperthyroidism, and Wilson’s disease, were evaluated and excluded based on normal blood glucose, thyroid function tests, and ceruloplasmin levels. Drug-induced and toxic causes were also considered. The patient had a history of corticosteroid use prior to the onset of symptoms; however, the neuropsychiatric disturbances and choreiform movements did not improve and progressively worsened after discontinuation, which argued against a corticosteroid-induced etiology. In addition, the patient denied exposure to any other medications or toxic substances. Among genetic and neurodegenerative causes, Huntington’s disease was considered, though the acute onset and lack of a relevant family history made this less likely. Finally, structural causes involving the basal ganglia were excluded based on the normal brain MRI findings. This systematic approach to the differential diagnosis ultimately guided the decision to pursue expanded neuronal antibody testing, which confirmed the diagnosis of anti-neurexin-3α autoimmune encephalitis.

Our case has several clinical implications. First, choreiform movements should be recognized as a possible prominent manifestation of neurexin-3α encephalitis, and neurologists should consider this antibody in patients presenting with acute/subacute psychiatric symptoms combined with hyperkinetic movement disorders. Second, normal or non-specific MRI does not rule out the diagnosis; CSF analysis and comprehensive antibody testing including neurexin-3α are essential. Third, tissue-based assay provides crucial directional value in patients with clinically suspected autoimmune encephalitis but negative conventional antibody screening, and should be actively utilized. Fourth, given the complex ICU course and concurrent rehabilitation, a comprehensive multi-modal immunotherapeutic strategy, including plasma exchange, FcRn antagonism, and targeted B-cell therapy, alongside vigorous rehabilitation, should be considered early in patients who fail to respond to first-line corticosteroids and intravenous immunoglobulin. Fifth, comprehensive ICU management and prevention of opportunistic infections in critically ill patients directly impact final outcomes.

Limitations: This is a single case report, and the proposed link between podocytopathy and neurexin-3α encephalitis through epitope spreading remains speculative. Further studies, including immunohistochemical analysis of renal biopsy specimens for neurexin family expression, longitudinal antibody titer monitoring, and larger cohort studies, are needed to confirm this hypothesis. Validated outcome scales such as MMSE, MoCA, modified Rankin Scale, or CASE were not applied at follow-up, which is a limitation of this case report. The favorable clinical outcome was assessed based on clinical presentation, normalization of follow-up EEG, and antibody titer dynamics, rather than on standardized scales.

## 4 Conclusion

In summary, this single case report highlights the complex clinical presentation and challenging management of neurexin-3α IgG-mediated autoimmune encephalitis. While the prominent choreiform movements observed in our patient may represent a noteworthy manifestation, the true prevalence and full spectrum of this phenotype warrant investigation in larger cohorts. The coexistence of biopsy-proven podocytopathy raises a hypothesis regarding shared antigenicity between renal podocyte and neuronal neurexin family members; however, this association remains purely speculative and does not imply a definitive causal link. Although EBV reactivation was observed in the cerebrospinal fluid, its role as a direct trigger or an epiphenomenon of severe immune dysregulation is yet to be determined.

Our experience suggests that a stepwise diagnostic approach, incorporating tissue-based assay when conventional antibody screening is negative, may provide crucial directional value. Early recognition, comprehensive immunotherapy, meticulous ICU management, and prevention of opportunistic infections appear to be important determinants of the favorable outcome in our patient. Furthermore, targeted agents such as efgartigimod and ofatumumab may represent novel therapeutic options for long-term maintenance; however, their specific efficacy and safety require validation in prospective studies. Given the inherent limitations of a single case report, these preliminary findings should be interpreted with caution and warrant confirmation through larger cohort studies and further mechanistic research.

## Data Availability

The original contributions presented in the study are included in the article/**Supplementary Material**. Further inquiries can be directed to the corresponding author.
