# Supplementary material for: Neurexin-3α IgG-mediated autoimmune encephalitis with psychiatric manifestations and choreiform movements: a case report and literature review
Source: Front Immunol. 2026 Oct 8;17:1944895. doi: 10.3389/fimmu.2026.1944895 (PMC13686998; doi:10.3389/fimmu.2026.1944895)
Supplement: Supplementary file 1 [file Table1.docx]

**CARE Checklist**

| Checklist Item | Location in Manuscript |
| --- | --- |
| **Title** – Neurexin-3α IgG-Mediated Autoimmune Encephalitis with Psychiatric Manifestations and Choreiform Movements: A Case Report and Literature Review | Title |
| **Key Words** – autoimmune encephalitis, neurexin-3α antibody, choreiform movements, podocytopathy, case report | Keywords |
| **Abstract** – (structured or unstructured) | Abstract |
| Introduction – What is unique about this case and what does it add to the scientific literature? | Abstract |
| The patient’s main concerns and important clinical findings. | Abstract |
| The primary diagnoses, interventions, and outcomes. | Abstract |
| Conclusion – What are one or more “take-away” lessons from this case report? | Abstract |
| **Introduction** – Briefly summarizes why this case is unique and may include medical literature references. | Introduction |
| **Patient Information** | Case Presentation |
| De-identified patient specific information. | Case Presentation |
| Primary concerns and symptoms of the patient. | Case Presentation |
| Medical, family, and psychosocial history including relevant genetic information. | Case Presentation |
| Relevant past interventions and their outcomes. | Case Presentation |
| **Clinical Findings** – Describe significant physical examination (PE) and important clinical findings. | Case Presentation |
| **Timeline** – Historical and current information from this episode of care organized as a timeline. | Case Presentation |
| **Diagnostic Assessment** |  |
| Diagnostic methods (PE, laboratory testing, imaging, surveys). | Case Presentation |
| Diagnostic challenges. | Discussion (Limitations) |
| Diagnosis (including other diagnoses considered). | Case Presentation; Discussion (Differential Diagnosis) |
| Prognostic characteristics when applicable. | N/A |
| **Therapeutic Intervention** |  |
| Types of therapeutic intervention (pharmacologic, surgical, preventive). | Case Presentation |
| Administration of therapeutic intervention (dosage, strength, duration). | Case Presentation |
| Changes in therapeutic interventions with explanations. | Case Presentation; Discussion |
| **Follow-up and Outcomes** |  |
| Clinician- and patient-assessed outcomes if available. | Case Presentation |
| Important follow-up diagnostic and other test results. | Case Presentation |
| Intervention adherence and tolerability. (How was this assessed?) | Case Presentation |
| Adverse and unanticipated events. | Case Presentation |
| **Discussion** |  |
| Strengths and limitations in your approach to this case. | Discussion |
| Discussion of the relevant medical literature. | Discussion |
| The rationale for your conclusions. | Discussion |
| The primary “take-away” lessons from this case report (without references) in a one paragraph conclusion. | Conclusion |
| **Patient Perspective** – The patient should share their perspective on the treatment(s) they received. | Case Presentation  patient reported full independence and satisfaction with recovery. |
| **Informed Consent** – The patient should give informed consent. (Provide if requested.) | Yes – Ethics Statement confirms written informed consent was obtained. |
